# Integrating 2D dosimetry and cell survival analysis for predicting local effect in spatially fractionated radiotherapy

**DOI:** 10.2340/1651-226X.2025.44599

**Published:** 2025-10-24

**Authors:** Delmon Arous, Jacob Larsen Lie, Nina Frederike Jeppesen Edin, Eirik Malinen

**Affiliations:** a Department of Physics, University of Oslo, Oslo, Norway; b Department of Medical Physics, Oslo University Hospital, Oslo, Norway; c Department of Radiation Biology, Oslo University Hospital, Oslo, Norway

**Keywords:** Neoplasms, radiotherapy, cell survival, models, biological, dose–response relationship

## Abstract

**Background and purpose:**

Robust methods for analysis and prediction of local cell survival after spatially fractionated radiotherapy (SFRT) *in vitro* remain limited. We present a methodology integrating spatial dosimetry with colony formation assessment and modelling to improve prediction of SFRT-induced responses.

**Patient/material and methods:**

A549 lung cancer cells were irradiated with 220 kV X-rays in three field patterns: open, striped, and dotted. Colony centroid locations were mapped from scanned images of culture flasks. Dose distributions were measured using radiochromic film dosimetry. Digital images with colony locations and dose maps were divided into 1 mm² quadrats. A Poisson regression model was fitted to colony counts per quadrat, incorporating linear-quadratic (LQ) model parameters α and β. A modified LQ (MLQ) model included an additional interaction between dose and nearest distance to a peak region, with parameter δ.

**Results:**

The methodology was successfully implemented. LQ fitting across all quadrats and patterns yielded α = 0.254 Gy^−^¹ and β = 0.039 Gy^−^², while the MLQ model gave α = 0.249 Gy^−^¹, β = 0.032 Gy^−^², and δ = −0.040 Gy^−^¹ cm^−^¹. Parameter uncertainty was below 0.5%. The MLQ model showed slightly lower fitting errors than the LQ model, indicating improved predictive accuracy.

**Interpretation:**

We introduce a novel analysis pipeline for 2D localization of colonies and SFRT survival modelling *in vitro*. Findings suggest that distance to peak dose regions significantly influences local SFRT effects. Incorporating this spatial factor via an MLQ model may enhance understanding and prediction of SFRT-induced survival.

## Introduction

In external beam radiation therapy, the conventional paradigm is to deliver a uniform, homogeneous radiation dose across the entire tumor volume while respecting the tolerance of surrounding healthy tissues. Spatially fractionated radiotherapy (SFRT) challenges this practice by intentionally delivering heterogeneous dose distributions, with high-dose ‘peaks’ and low-dose ‘valleys’, with the aim to increase the therapeutic index [1, 2]. The SFRT approach has been used clinically for decades, mainly for the palliative treatment of large, bulky tumors, and has in some cases led to tumor control [3–6]. SFRT has gained renewed interest due to an improved understanding of how its effects go beyond direct cell death, involving non-targeted responses like radiation-induced bystander effects (RIBEs) and immune activation [7, 8]. New clinical findings are also emerging [9–11].

GRID therapy is a two-dimensional (2D) SFRT technique where radiation is delivered through an array of beamlets, creating a grid pattern. For megavoltage photons, this pattern is typically generated using commercial brass collimators attached to a linear accelerator or by using a multileaf collimator (MLC). Dosimetrically, the dose distribution is characterized by the beamlet width, the center-to-center spacing, and the resulting peak-to-valley dose ratio (PVDR) [1, 12]. Clinical application of GRID therapy typically involves delivering a peak dose of 15–20 Gy [12]. A review of preclinical SFRT studies highlighted that the valley dose, rather than the peak dose, correlates best with treatment outcome [13]. Nevertheless, peak doses are still considered crucial for cytotoxic cell killing and for triggering secondary radiobiological processes, such as immune cell activation. It is therefore important to map the entire dosimetric pattern, as both peak and valley regions are determinantal for cell viability and biological response.

Assessing the local biological effects from GRID irradiation is challenging. Establishing a robust pipeline for quantifying in vitro responses, which includes precise mapping of radiation dose and cell survival, could facilitate improved biological response modeling. Various mathematical models have been derived which take into account cellular effects beyond direct DNA damage, such as the diffusion of signaling factors [14–16]. It is therefore pivotal to have a framework that can experimentally validate such candidate models using clonogenic survival data. With that, the purpose of the presented work was to analyze and predict clonogenic survival in vitro using a novel 2D quadrat Poisson regression approach incorporating a generalized radiobiological model. The analysis was performed with two different X-ray GRID irradiation patterns in addition to homogeneous (open) irradiation. The developed framework is intended to support the assessment of the biological effects of GRID therapy and help in bridging physical dose parameters and cellular response.

## Material and methods

### Clonogenic assay

The A549 human lung adenocarcinoma cell line was used to assess clonogenic survival following irradiation. Cells were seeded at a density of 10,000 cells per T25 flask (Nunclon, Denmark), incubated, and allowed to adhere for 24 h. To eliminate artifacts related to cell density, a consistent seeding number was maintained across all experiments, resulting in nearly invariant plating efficiencies (PEs). The PE, estimated using an in-house colony segmentation algorithm (see below), was approximately 63%. Following irradiation (see below), the flasks were incubated for ~6 days to allow colony formation. Colonies were then fixed and stained using 96% technical ethanol (Antibac, Norway) and Methylene Blue (Sigma, USA). The stained T25 flasks were imaged and analyzed using a custom machine learning-based colony segmentation method to quantify clonogenic survival [17,18].

### Irradiation setup

**Figure 1:**
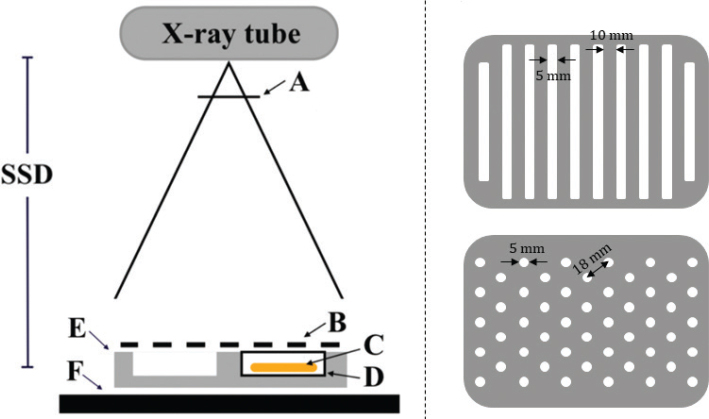
(Left) Schematic of the experimental setup for GRID irradiation: X-ray filtration (A), tungsten grid collimator (B), EBT3 film and A549 cells in culture medium (C), T25 cm² cell culture flask (D), polymethyl methacrylate (PMMA) flask holder (E), and Perspex table (F). (Right) Beam’s-eye view of the striped (top) and dotted (bottom) tungsten grid collimators.

Both open-field (homogeneous, uncollimated) and GRID (heterogeneous, collimated) X-irradiation were performed using a Pantak PMC 1000 X-ray unit (Pantak, USA). The X-ray settings were 220 kV tube voltage, 10 mA current, with a 0.70 mm copper and 1.52 mm aluminum filter. The source-to-surface distance (SSD) was 58 cm, measured to the surface of the cell culture medium in the T25 flasks. GRID irradiation was achieved using custom-made tungsten collimators to create either striped or dotted dose patterns (see Figure 1). The striped GRID pattern was generated using a collimator with periodic 5 mm open and 10 mm blocked regions. The dotted pattern employed a collimator with 5 mm diameter circular openings, spaced 18 mm center-to-center. Open-field irradiation was delivered without collimation. Each collimator was mounted on a custom-built polymethyl methacrylate (PMMA) cell flask holder, designed with four cavities to accommodate T25 flasks. The holder rested on a Perspex plate inside the irradiation chamber. To maintain physiological temperature, the Perspex plate was preheated by circulating air to ensure 37°C in the cell culture medium during irradiation. Single doses of 2, 5, and 10 Gy were delivered, with four replicates per dose and field configuration, in addition to four unirradiated controls. The reported doses represent actual absorbed doses for the open-field configuration and nominal doses for the GRID irradiation patterns.

### Radiochromic film dosimetry

Gafchromic™ EBT3 films were used to measure the 2D dose distribution in the T25 cell culture flasks. EBT3 films (lot No. 02122001) were processed according to the recommended guidelines issued by AAPM Task Group 235 for film handling and scanning [19]. The films were positioned and irradiated identically to the A549 cells (see Figure 1), ensuring a 1:1 mapping between delivered dose and clonogenic survival. Prior to EBT3 film irradiation, reference dosimetry in an open field was performed using a Scanditronix-Wellhöfer FC65-G Farmer-type ionization chamber (IBA Dosimetry, Schwarzenbruck, Germany) to determine the dose rate. The absorbed dose to water was determined by irradiating the ionization chamber placed inside an empty T25 flask. The measured dose rate was 0.59 ± 0.02 Gy/min at a representative distance matching the location of the cell flasks and films.

For calibration, EBT3 film sheets were cut into smaller pieces fitting the cell flasks (6.5 × 4.3 cm²), and eight film pieces were used per nominal dose to mitigate inter-film variation. Using an open field setup, the films were irradiated to doses of 0, 0.1, 0.2, 0.5, 1, 2, 5, and 10 Gy. All films were covered with 1 mm PMMA and irradiated inside empty T25 flasks. For GRID dosimetry, a nominal dose of 5 Gy was delivered to 16 film pieces per field pattern. Dose maps corresponding to 2 and 10 Gy were subsequently derived through relative scaling. The film dose response was quantified 48 h post-irradiation using an EPSON^®^ Perfection V850 Pro flatbed scanner and Scan software (Epson Seiko Corp., Nagano, Japan). All film pieces were positioned in a fixed central region on the scanner bed and scanned in identical orientation to minimize positional variation. Image acquisition was performed in transmission and RGB-positive mode at a spatial resolution of 300 dpi and 16-bit color depth.

All image data were processed using MATLAB 2022a (MathWorks, Natick, MA, USA). As previously described [17], in-house scripts were used to analyze the digitized images in all three color channels (red, green, blue). For each calibration film, a 4 × 4 mm² region of interest (ROI) was sampled at the image center to extract the mean of pixel intensity. The film response to dose was defined as the net optical density (netOD) change in each ROI, corrected for background signal. The relationship between netOD and absorbed dose was determined via the calibration films. A suitable functional model was fitted to the netOD data using least-squares regression, as described previously [17]. Calibration curves were generated for each channel (red, green, blue), and model performance was evaluated by the coefficient of determination (*R*²) and root-mean-square error (RMSE). While all fits were statistically significant (data not shown), the red channel consistently yielded the highest R² and lowest RMSE and was therefore selected for estimating the 2D dose distribution in the cell flasks.

### Cell colony localization

We have previously developed an image segmentation technique based on a principal component–based watershed method to identify individual colonies within a cell flask [18]. This algorithm was applied to scanned images of flasks containing fixed and stained A549 cell colonies to localize the centroids of surviving colonies, using preset parameters optimized for this cell line and a supervised calibration procedure [17]. The image dataset from the colony assay was acquired using the flatbed scanner and scanning protocol described earlier in the text, with a spatial resolution of 1,200 dpi. To ensure consistency, the cell flasks were positioned and scanned at the same location on the scan surface as the EBT3 films. Through this approach, the spatial dose distribution obtained from film dosimetry was directly mapped onto the locations of surviving colonies, enabling a one-to-one correspondence between delivered dose and clonogenic outcome.

### Poisson regression analysis

**Figure 2:**
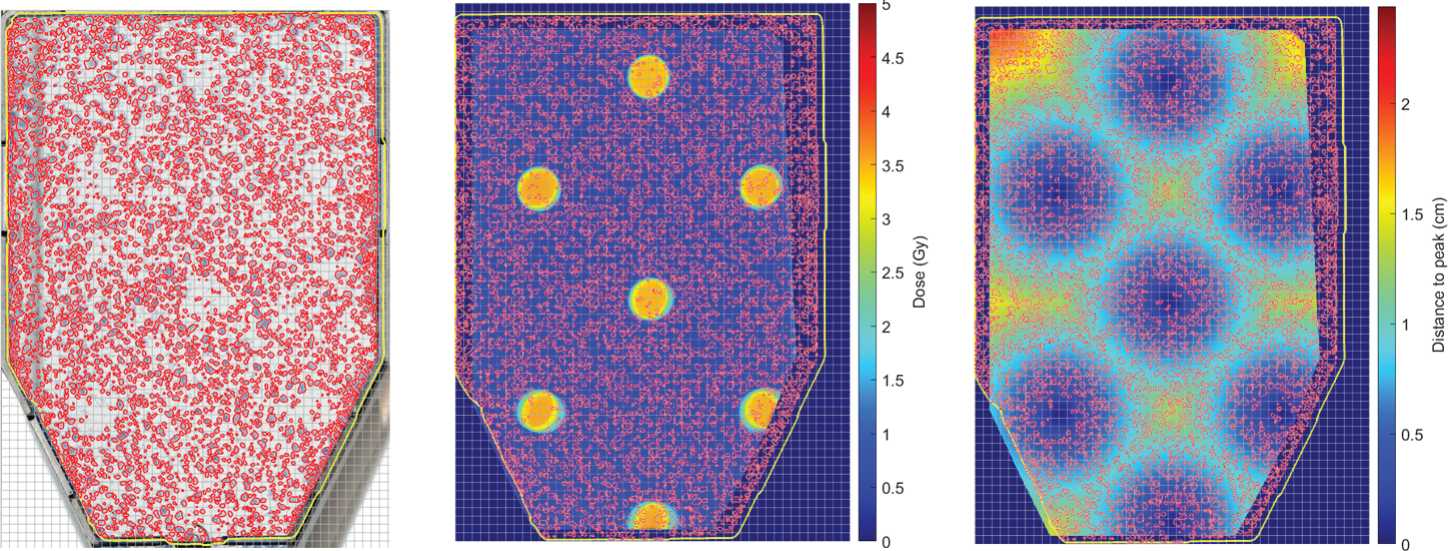
(Left) Segmented A549 colonies (red outlines) in a flask irradiated with a dotted field at a nominal dose of *D* = 5 Gy. The overlaid grey mesh divides the image into 1 mm² quadrats. (Centre) Spatially co-registered dose distribution within the flask, with the colony contours and flask outline superimposed. (Right) Euclidean distance map showing the distance from each point in the flask to the nearest rim of a peak region, with the colony contours and flask outline superimposed.


With the spatial alignment of cell colonies and radiation dose, we were able to assess the local dose response following both open and GRID irradiation fields. Using the dose maps, the shortest Euclidean distance *d* from each pixel to the rim of the nearest peak region was computed for each GRID pattern. The peak region was defined as being above 95% of maximum dose for a given GRID configuration. The digital images containing colony centroids, radiation dose, and distance to the peak were paired, co-registered, and subsequently divided into smaller blocks (quadrats) for Poisson regression analysis (Figure 2). A quadrat size of 1 mm² was chosen, partitioning the images into 64 × 47 quadrats, with each quadrat consisting of 47 × 47 pixels. Only quadrats entirely contained within the overlapping region of both the cell flask and dose film were included in the analysis. Within each quadrat, the number of colonies *NC* was counted from the segmented flask images. The dose map from film dosimetry provided the local quadrat dose *D*. Furthermore, we employed the linear-quadratic (LQ) model:

$$NC_{LQ}=NC_{0}e^{–\alpha D–\beta D^{2}}=e^{\lambda_{0}–\alpha D–\beta D^{2}}$$ (1)

where *NC*_0_ is the expected number of colonies at zero dose, ${\text{λ}}_{\text{0}}=\text{ln}\left(NC_{\text{0}}\right)$, and *α* and *β* are the linear and quadratic cell kill coefficients, respectively. Since *NC* is a discrete random variable assumed to follow the Poisson distribution, conventional least squares regression is not statistically appropriate for estimating the coefficients. As seen, Eq. (1) is a variant of a Poisson generalized linear model [20]:


lnλ=Χξ (2)

where *λ* is the expected event rate, *Χ* the matrix of regression variables, and *ξ* the vector of regression coefficients. Using count statistics from each quadrat and their corresponding doses, we fitted Eq. (1) via MATLAB’s glmfit function to quadrat counts and doses to estimate $NC_{0}\left(=e^{\lambda 0}\right)$, *α* and *β*.

To tentatively account for non-targeted effects in GRID irradiation, we extended the LQ model into a modified linear-quadratic (MLQ) form:


$${NC}_{\text{MLQ}}={NC}_{0}e^{-\alpha D-\beta D^{2}-\delta Dd}$$ (3)

Here, the term *δDd* represents a bystander effect arising from intercellular signaling, where cells in valley regions may receive signals from irradiated peak regions. The variable d modulates this interaction by quantifying the distance from the nearest peak. This term vanishes in peak regions (*d* = 0) and for open fields. A negative *δ* indicates beneficial (‘survival’) signals to valley cells, while a positive *δ* indicates harmful (‘damage’) signals.

The dataset comprised T25 cell flasks irradiated with nominal doses of 2, 5, and 10 Gy, with four replicates per dose per irradiation pattern (open, striped, dotted), plus four unirradiated controls. We employed four-fold cross-validation (CV), using 75% of the data for training and 25% for testing. All colony quadrat data (control, open, striped, dotted) were analyzed jointly for each model and fold. Model performance was evaluated using the Akaike Information Criterion (AIC) and Bayesian Information Criterion (BIC). Additionally, RMSE and mean absolute error (MAE) were used to assess predictive accuracy. The CV procedure was repeated 4,000 times to generate 95% confidence intervals (CIs) for all model fit parameters, AIC, BIC, RMSE, and MAE. To test for statistically significant differences in predicted colony counts between the LQ and MLQ models for each nominal dose, we performed two-tailed Student’s *t*-tests. A *p*-value ≤ 0.05 was considered statistically significant.

## Results

**Figure 3:**
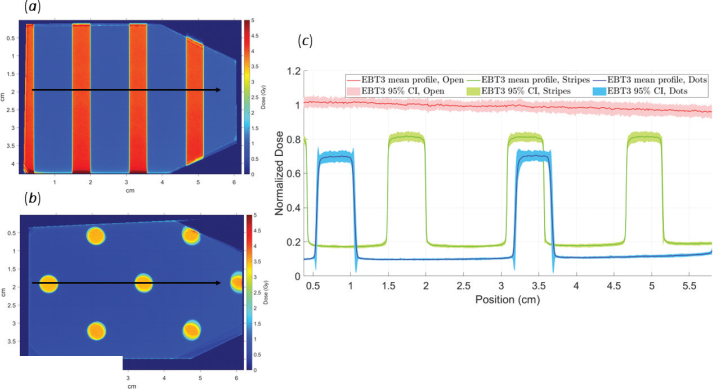
2D film dosimetry results for striped (A) and dotted (B) irradiation. The dose profiles (C) show normalized doses for open (red), striped (green), and dotted (blue) fields, with shaded regions representing the 95% confidence intervals.

Central dose profiles from the film measurements are shown in Figure 3. As observed, both the striped and dotted field deliveries result in lower peak doses compared to the open field. At a nominal dose of 5 Gy, the films exposed to a striped field received an average peak dose of 4.1 Gy and a valley dose of 0.9 Gy, while those exposed to a dotted field received 3.5 Gy peak and 0.5 Gy valley doses, respectively (Table 1). Thus, with striped and dotted fields, approximately 18 and 30% of the dose is lost in the peak regions relative to open field irradiation. Additionally, about 22 and 14% of the peak dose is scattered into the valleys for the striped and dotted fields, respectively. Overall, the mean PVDRs were estimated from the film dosimetry to be 4.7 (95% CI: 4.5–4.9) for striped fields and 7.0 (95% CI: 6.7–7.4) for dotted fields.

**Table 1:** Mean dose estimates from EBT3 films following open, striped, and dotted irradiation together with 95% CIs estimated from film dosimetry.

Open dose (Gy)	Striped Peak dose (Gy), Valley dose (Gy)	Dotted Peak dose (Gy), Valley dose (Gy)
5.0 (4.8, 5.1)	4.1 (3.9, 4.2), 0.9 (0.8, 1.0)	3.5 (3.4, 3.7), 0.5 (0.4, 0.6)

**Figure 4:**
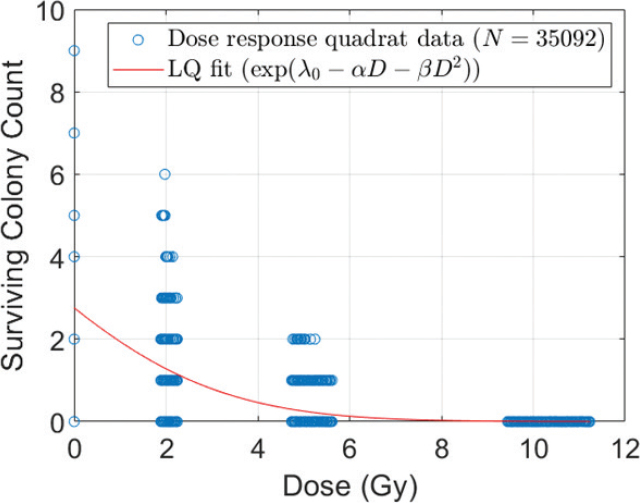
Colony count in local quadrats as a function of dose for open field irradiation. The solid line represents the LQ model fitted through Poisson regression on measured quadrat data (open circles).

**Figure 5:**
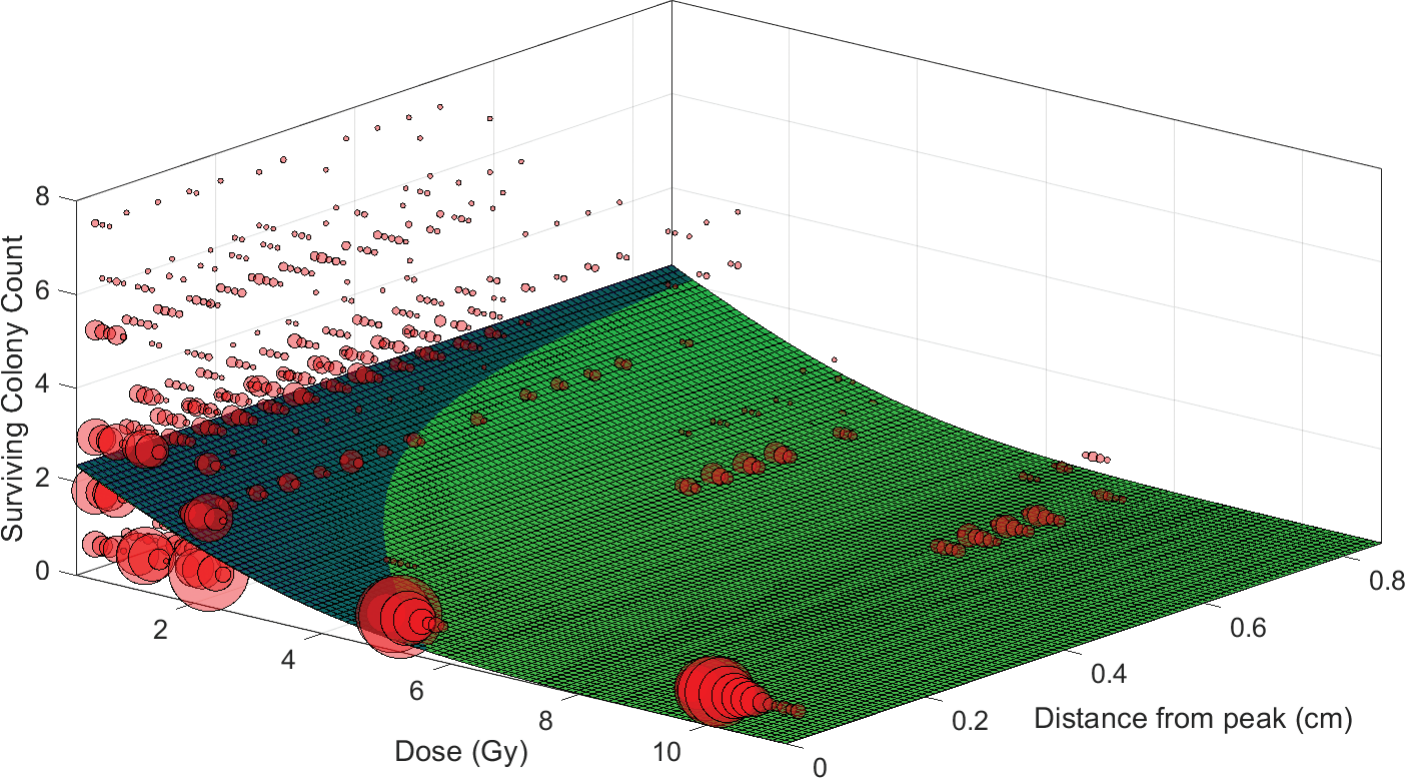
Surface plot comparing the LQ model (green) and the MLQ model (blue), both fitted with Poisson regression to quadrat-level colony count data (filled circles). Colony counts were modelled against mean quadrat dose (LQ and MLQ) and distance to the nearest peak region (MLQ). Models were trained and tested with repeated four-fold cross-validation; the data shown are from the first fold.

Colony counts per quadrat were collected for all cell flasks and field configurations along with their corresponding local dose values. Chi-square tests indicated that the count data were consistent with a Poisson distribution, showing no evidence of overdispersion or zero-inflation (data not shown). To illustrate the methodology, we first applied the analysis framework to open field irradiation, where the Poisson regression yielded the following parameter estimates: *NC*_0_ = 2.77 ± 0.03, *α* = 0.32 ± 0.01 Gy^−1^ and *β* = 0.031 ± 0.002 Gy^−2^. As shown in Figure 4, the quadrat-based approach inherently accounted for spatial dose heterogeneity in the delivered open field, enabling a more refined dose–response analysis. Furthermore, by modeling colony counts as Poisson-distributed variables, the method appropriately reflected the discrete and stochastic nature of the data, thereby providing statistically robust estimates of the cell survival model parameters. Using the quadrat-based Poisson regression framework, all colony count data – spanning open, striped, and dotted field configurations – were jointly analyzed using four-fold CV. The resulting model parameters for both the LQ and MLQ models are presented in Table 2. All estimated parameters were found to be statistically significant (*p* ≤ 0.001), indicating the robustness of the fits. Although the improvements were modest, both the AIC and BIC favored the MLQ model over the conventional LQ model, suggesting a better overall model fit when including the additional spatial variable. Further evaluation using RMSE and MAE metrics on the validation data confirmed the slight performance advantage for the MLQ model. Figure 5 displays surface plots of the LQ and MLQ fits over the observed quadrat data. Both models exhibit similar behavior at higher doses – corresponding to peak regions in the GRID configurations. However, notable differences emerge at lower doses: the MLQ model predicts increased colony survival relative to the LQ model. This can be attributed to the estimated δ coefficient being negative, indicating a potential pro-survival or protective bystander effect in valley regions.

**Table 2:** Estimates for training (fitting) and validation (testing) of the LQ and MLQ models. The estimated model parameter and error metric values are presented as means together with 95% CIs.

		LQ model	MLQ model
Training	NC_0_	2.4480 (2.4476, 2.4483)	2.4719 (2.4717, 2.4722)
	α (Gy^–1^)	0.2539 (0.2538, 0.2541)	0.2485 (0.2482, 0.2487)
	β (Gy^–2^)	0.0388 (0.0387, 0.0389)	0.0321 (0.0318, 0.0324)
	δ (Gy^–1^ cm^–1^)	–	–0.0401 (–0.0403, –0.0400)
	AIC	112411 (112408, 112412)	112403 (112400, 112405)
	BIC	112438 (112436, 112439)	112437 (112434, 112439)
Validation	RMSE	1.0053 (1.0052, 1.0054)	1.0050 (1.0049, 1.0051)
	MAE	0.7070 (0.7069, 0.7072)	0.7039 (0.7037, 0.7040)

RMSE: root-mean-square error; AIC: Akaike Information Criterion; BIC: Bayesian Information Criterion; MAE: mean absolute error.

## Discussion and conclusion

It is imperative to have robust methods for accurately quantifying and predicting local cell survival in vitro following SFRT. To our knowledge, few studies have attempted to quantify these local effects in a stringent and generalized manner. In this work, we have presented a comprehensive pipeline to analyze in vitro GRID effects, including different irradiation patterns, automated colony localization, film dosimetry with 1:1 mapping of colonies to radiation dose, and quadrat-based dose-response analysis using Poisson generalized linear regression models. Our new approach enabled a deeper appraisal of local effects from GRID dose delivery, tentatively introducing a coefficient to explain a pro-survival effect observed for cells located in the valley regions.

In general, the survival of a cell in a non-uniform radiation field depends on its location. In this scenario, local effect assessment requires analyzing small regions of a cell flask, which often contain a limited number of colonies, resulting in Poisson-distributed data. Therefore, Poisson generalized linear regression is a more statistically appropriate method of analysis than conventional least squares regression, which assumes Gaussian-distributed data. Facilitated by our methodological pipeline, we introduced the MLQ model to appraise ‘damage’ or ‘survival’ signals potentially transmitted from cells in high-dose peaks to bystander cells in the valleys. Our goal was not to introduce a unifying model for all RIBE phenomena, but rather to demonstrate the power of our established methodology to test such hypotheses.

Several methodologies have been employed in the literature to analyze GRID effects in cell cultures, making direct comparisons between studies challenging. One approach involves physically harvesting cells from distinct peak and valley regions after irradiation, followed by separate replating for each bulk population [21, 22]. Furthermore, a method proposed by Peng et al. involves mathematical modeling, where models that include bystander terms are fitted to average cell survival data across the entire GRID-irradiated culture flask to estimate a parameter representing the overall bystander effect [15,16]. The fitted models could subsequently be projected onto observed survival profiles. In contrast, our presented pipeline offers a more detailed analysis by combining high-resolution 2D dose mapping with the precise localization of every surviving colony. By partitioning the inhomogenously irradiated culture into small quadrats and applying Poisson regression, our framework directly models local colony counts as a function of both local dose and spatial parameters. While comparing outcomes across these different analytical strategies is difficult due to variations in experimental setups and cell lines, our methodology is arguably the most advanced to date. It avoids the spatial averaging inherent in bulk harvesting with subsequent replating or average survival analyses, providing a more stringent and statistically robust framework to quantify local radiobiological responses and validate models of intercellular signaling in spatially complex radiation fields.

A key finding of our study is that the negative δ coefficient in the MLQ model indicated that valley cells receive transmitted signals from peak cells that enhance local survival. The nature of bystander effects is complex, with reports of both pro-survival and cytotoxic (cell-killing) outcomes in the literature [8, 23]. For instance, Asur et al. [21] performed GRID experiments on murine carcinoma cells and observed a significant cytotoxic bystander effect, where survival in the valley regions was lower than expected from the scatter dose alone. Similarly, Peng et al. [15], in testing several bystander models, found that modulated fields produced a lower survival fraction than uniform fields of the same average dose, which is also consistent with a net cytotoxic bystander effect. Our finding of a pro-survival signal for valley cells reveals a different aspect of this phenomenon, although no studies have assessed the SFRT response in a similar manner. These differences highlight the fact that the bystander response is not universally cytotoxic, as has also been discussed previously [8, 23]. The specific direction of the bystander effect is likely cell-line specific and can be heavily influenced by experimental conditions like seeding density and incubation time, which affect the release and concentration of molecular signaling factors.

The introduced MLQ model, with its additional spatial explanatory variable, marginally outperforms the conventional LQ model in predicting average cell survival, a conclusion substantiated by lower AIC, BIC, RMSE, and MAE values. This finding is in line with a previous modelling study concluding that models incorporating a bystander term were superior to the classical LQ model in predicting cell survival in modulated radiation fields [15]. While we introduced the δ term in the MLQ model as a proxy for spatially mediated bystander signaling, we acknowledge that it may also capture other factors inherent to GRID irradiation. In particular, valleys experience not only lower cumulative dose but also lower instantaneous dose rates, which can facilitate sublethal damage repair during exposure and thereby contribute to apparent survival benefits. Our present data do not allow us to disentangle these mechanisms, and we therefore interpret δ more broadly as an empirical descriptor of spatially modulated survival effects. Nonetheless, the negative δ coefficient observed here is consistent with a pro-survival contribution, tentatively supporting the general hypothesis that RIBEs in SFRT can be mediated by extracellular soluble factors. Future studies that combine functional assays of intercellular communication with controlled manipulations of dose rate will be required to distinguish genuine signaling effects from dose–rate–dependent repair processes.

In conclusion, we have introduced an advanced pipeline for mapping surviving cell colonies following GRID irradiation and modeling the local survival data using Poisson regression. Using this framework, we found that the conventional LQ model was less capable of predicting survival than the developed MLQ model, which accounts for spatial dose heterogeneity. Through the MLQ model, we determined that local dose was not the only significant predictor of cell survival in modulated fields; the distance from high-dose peak regions also plays a role, suggesting a bystander survival signal mediated by diffusible factors. This work provides a robust framework for quantifying such effects and contributes to the understanding of the complex radiobiology of SFRT.

## Data Availability

The datasets generated and analyzed during the current study are available from the corresponding author upon reasonable request.

## References

[1] Prezado Y, Grams M, Jouglar E, Martínez-Rovira I, Ortiz R, Seco J, et al. Spatially fractionated radiation therapy: a critical review on current status of clinical and preclinical studies and knowledge gaps. Phys Med Biol. 2024;69(10):10TR02. 10.1088/1361-6560/ad4192 38648789

[2] Yan W, Khan MK, Wu X, Simone CB, 2nd, Fan J, Gressen E, et al. Spatially fractionated radiation therapy: history, present and the future. Clin Transl Radiat Oncol. 2020;20:30–8. 10.1016/j.ctro.2019.10.004 31768424 PMC6872856

[3] Laissue JA, Blattmann H, Slatkin DN. [Alban Kohler (1874–1947): inventor of grid therapy]. Z Med Phys. 2012;22(2):90–9. 10.1016/j.zemedi.2011.07.002 21862299

[4] Marks H. Clinical experience with irradiation through a grid. Radiology. 1952;58(3):338–42. 10.1148/58.3.338 14900413

[5] Mohiuddin M, Fujita M, Regine WF, Megooni AS, Ibbott GS, Ahmed MM. High-dose spatially-fractionated radiation (GRID): a new paradigm in the management of advanced cancers. Int J Radiat Oncol Biol Phys. 1999;45(3):721–7. 10.1016/S0360-3016(99)00170-4 10524428

[6] Neuner G, Mohiuddin MM, Vander Walde N, Goloubeva O, Ha J, Yu CX, et al. High-dose spatially fractionated GRID radiation therapy (SFGRT): a comparison of treatment outcomes with Cerrobend vs. MLC SFGRT. Int J Radiat Oncol Biol Phys. 2012;82(5):1642–9. 10.1016/j.ijrobp.2011.01.065 21531514

[7] Daguenet E, Louati S, Wozny AS, Vial N, Gras M, Guy JB, et al. Radiation-induced bystander and abscopal effects: important lessons from preclinical models. Br J Cancer. 2020;123(3):339–48. 10.1038/s41416-020-0942-3 32581341 PMC7403362

[8] Jenkins SV, Johnsrud AJ, Dings RPM, Griffin RJ. Bystander effects in spatially fractionated radiation therapy: from molecule to organism to clinical implications. Semin Radiat Oncol. 2024;34(3):284–91. 10.1016/j.semradonc.2024.05.004 38880537 PMC11185274

[9] Knight JA, 2nd, Trosper N, Misa J, Bernard ME, Fabian D, Kudrimoti M, et al. Reported early clinical outcomes of forward-planned multileaf collimator-based 3-dimensional conformal spatially fractionated radiation therapy technique for large and Bulky tumors. Int J Radiat Oncol Biol Phys. 2025;122(4):1060–8. 10.1016/j.ijrobp.2025.04.016 40298857

[10] Xu P, Wang S, Zhou J, Yuan K, Wang X, Li L, et al. Spatially fractionated radiotherapy (Lattice SFRT) in the palliative treatment of locally advanced bulky unresectable head and neck cancer. Clin Transl Radiat Oncol. 2024;48:100830. 10.1016/j.ctro.2024.100830 39219705 PMC11364051

[11] Grams MP, Mateus CQ, Mashayekhi M, Mutter RW, Djonov V, Fazzari JM, et al. Minibeam radiation therapy treatment (MBRT): commissioning and first clinical implementation. Int J Radiat Oncol Biol Phys. 2024;120(5):1423–34. 10.1016/j.ijrobp.2024.06.035 39002850

[12] Ahmed MM, Wu X, Mohiuddin M, Perez NC, Zhang H, Amendola BE, et al. Optimizing GRID and lattice spatially fractionated radiation therapy: innovative strategies for radioresistant and Bulky tumor management. Semin Radiat Oncol. 2024;34(3):310–22. 10.1016/j.semradonc.2024.05.002 38880540

[13] Fernandez-Palomo C, Chang S, Prezado Y. Should peak dose be used to prescribe spatially fractionated radiation therapy? – A review of preclinical studies. Cancers. 2022;14(15):3625. 10.3390/cancers14153625 35892895 PMC9330631

[14] Cahoon P, Giacometti V, Casey F, Russell E, McGarry C, Prise KM, et al. Investigating spatial fractionation and radiation induced bystander effects: a mathematical modelling approach. Phys Med Biol. 2021;66(22):225007. 10.1088/1361-6560/ac3119 34666318

[15] Peng V, Suchowerska N, Esteves ADS, Rogers L, Claridge Mackonis E, Toohey J, et al. Models for the bystander effect in gradient radiation fields: Range and signalling type. J Theor Biol. 2018;455:16–25. 10.1016/j.jtbi.2018.06.027 30008397

[16] Peng V, Suchowerska N, Rogers L, Claridge Mackonis E, Oakes S, McKenzie DR. Grid therapy using high definition multileaf collimators: realizing benefits of the bystander effect. Acta Oncol. 2017;56(8):1048–59. 10.1080/0284186X.2017.1299939 28303745

[17] Arous D, Lie JL, Hsland BV, Borsting M, Edin NFJ, Malinen E. 2D mapping of radiation dose and clonogenic survival for accurate assessment of in vitro X-ray GRID irradiation effects. Phys Med Biol. 2023;68(2):025024. 10.1088/1361-6560/acaf20 36580679

[18] Arous D, Schrunner S, Hanson I, Edin NFJ, Malinen E. Principal component-based image segmentation: a new approach to outline cell colonies. Comput Methods Biomech Biomed Eng-Imaging Visual. 2023;11(1):18–30. 10.1080/21681163.2022.2035822

[19] Niroomand-Rad A, Chiu-Tsao ST, Grams MP, Lewis DF, Soares CG, Van Battum LJ, et al. Full report of AAPM Task Group 235 radiochromic film dosimetry: an update to TG-55. Med Phys. 2020;47(12):5986–6025. 10.1002/mp.14497 32990328

[20] Roback P, Legler J. Beyond multiple linear regression: applied generalized linear models and multilevel models in R. Boca Raton, FL. Chapman & Hall/CRC; 2024.

[21] Asur RS, Sharma S, Chang CW, Penagaricano J, Kommuru IM, Moros EG, et al. Spatially fractionated radiation induces cytotoxicity and changes in gene expression in bystander and radiation adjacent murine carcinoma cells. Radiat Res. 2012;177(6):751–65. 10.1667/RR2780.1 22559204 PMC3395590

[22] Butterworth KT, McMahon SJ, McKee JC, Patel G, Ghita M, Cole AJ, et al. Time and cell type dependency of survival responses in co-cultured tumor and fibroblast cells after exposure to modulated radiation fields. Radiat Res. 2015;183(6):656–64. 10.1667/RR13992.1 25973952

[23] Asur R, Butterworth KT, Penagaricano JA, Prise KM, Griffin RJ. High dose bystander effects in spatially fractionated radiation therapy. Cancer Lett. 2015;356(1):52–7. 10.1016/j.canlet.2013.10.032 24246848 PMC4022709

